# 700 Implementation of an Intranasal Decolonization Protocol and Line Changing Policy in Adult Burn Patients

**DOI:** 10.1093/jbcr/irad045.176

**Published:** 2023-05-15

**Authors:** Adalah Yahia, Katie Barber, Shelbye Herbin, Michael White, Janie Faris, Lester Laddaran, Katherine Kowalski

**Affiliations:** Detroit Receiving Hospital, Melvindale, Michigan; University of Mississippi School of Pharmacy, Jackson, Mississippi; Henry Ford Health, Ferndale, Michigan; Detroit Medical Center, Detroit, Michigan; Parkland, Grapevine, Texas; Detroit Medical Center, Detroit, Michigan; Wayne Health, Detroit, Michigan; Detroit Receiving Hospital, Melvindale, Michigan; University of Mississippi School of Pharmacy, Jackson, Mississippi; Henry Ford Health, Ferndale, Michigan; Detroit Medical Center, Detroit, Michigan; Parkland, Grapevine, Texas; Detroit Medical Center, Detroit, Michigan; Wayne Health, Detroit, Michigan; Detroit Receiving Hospital, Melvindale, Michigan; University of Mississippi School of Pharmacy, Jackson, Mississippi; Henry Ford Health, Ferndale, Michigan; Detroit Medical Center, Detroit, Michigan; Parkland, Grapevine, Texas; Detroit Medical Center, Detroit, Michigan; Wayne Health, Detroit, Michigan; Detroit Receiving Hospital, Melvindale, Michigan; University of Mississippi School of Pharmacy, Jackson, Mississippi; Henry Ford Health, Ferndale, Michigan; Detroit Medical Center, Detroit, Michigan; Parkland, Grapevine, Texas; Detroit Medical Center, Detroit, Michigan; Wayne Health, Detroit, Michigan; Detroit Receiving Hospital, Melvindale, Michigan; University of Mississippi School of Pharmacy, Jackson, Mississippi; Henry Ford Health, Ferndale, Michigan; Detroit Medical Center, Detroit, Michigan; Parkland, Grapevine, Texas; Detroit Medical Center, Detroit, Michigan; Wayne Health, Detroit, Michigan; Detroit Receiving Hospital, Melvindale, Michigan; University of Mississippi School of Pharmacy, Jackson, Mississippi; Henry Ford Health, Ferndale, Michigan; Detroit Medical Center, Detroit, Michigan; Parkland, Grapevine, Texas; Detroit Medical Center, Detroit, Michigan; Wayne Health, Detroit, Michigan; Detroit Receiving Hospital, Melvindale, Michigan; University of Mississippi School of Pharmacy, Jackson, Mississippi; Henry Ford Health, Ferndale, Michigan; Detroit Medical Center, Detroit, Michigan; Parkland, Grapevine, Texas; Detroit Medical Center, Detroit, Michigan; Wayne Health, Detroit, Michigan

## Abstract

**Introduction:**

Reducing infection in burn patients can have a major impact on clinical outcomes. The purpose of this study is to evaluate the rate of bacteremia pre/post the implementation of a line change and nasal decolonization protocol.

**Methods:**

This quasi-experimental study assessed adult patients admitted to a single center ABA verified burn center from August 2019 - August 2020 (pre-intervention group) versus September 2020 - September 2021 (post-intervention group). Patients must have received at least 1 round of intranasal decolonization treatment (mupirocin intranasal twice a day for 5 days and repeated every 30 days during hospital stay) during the post-protocol period. A line change policy was also implemented post protocol period for central and peripheral lines. Patients were excluded if they had a total loss of nasal alae, injury deemed nonsurvivable, or transitioned to comfort care within 72 hours of admission.

**Results:**

A total of 558 burn patients were included, 324 patients in the pre-group and 234 post-group. There was no significant difference in comorbidities between the two groups. The majority of patients were admitted for an integumentary burn (87.6%) with the most common cause being flame (49.8%) or hot water contact (19.0%). Compliance with the decolonization protocol was appropriate for 87.6% of patients post-intervention. During the pre-intervention period, there were 18 (7.7%) positive blood cultures versus only 9 (3.8%) in the post-group (p=0.426). Gram-positive infections occurred in 44.4% of the pre-group and only 11.1% in the post-group (p=0.087). The rate of Gram-negative infections was similar between the pre and post-groups at 44.4% and 55.6%, p = 0.695. MRSA bacteremia was 44.4% of total Gram-positive bacteremia, with pre and post-intervention 4 (50%) vs. 0 (0%), p = 0.1. The median time of line placement to bacteremia pre-intervention was 7(5-12) days vs. post-intervention 11(6-14) days (p = 0.220). Hospital mortality between both periods was 9(3.8%) vs. 5(2.1%), p = 0.786. No significant difference was found in hospital LOS, ICU LOS, or discharge disposition.

**Conclusions:**

Overall, this study showed a 50% decreased rate of MRSA bacteremia with the implementation of an intranasal decolonization protocol and a delay in Gram-negative bacteremia post implementation of a line change policy, potentially leading to a decrease in morbidity and hospital bloodstream infection-related costs.

**Applicability of Research to Practice:**

Reducing infection rates can have a major impact on burn patient outcomes and lead to significant cost savings.

